# Distribution interactions of the trace elements zinc, copper, and selenium under conditions of their parallel deficiency

**DOI:** 10.1016/j.redox.2025.103963

**Published:** 2025-12-06

**Authors:** Kristina Lossow, Maria Maares, Tom Heinze, Denny Pellowski, Elisa Richter, Karolin Schröder, Lars Dahmen, Christoph Schüßler, Kostja Renko, Tanja Schwerdtle, Hajo Haase, Anna P. Kipp

**Affiliations:** a Department of Nutritional Physiology, Institute of Nutritional Sciences, Friedrich Schiller University Jena, Jena, Germany; b TraceAge-DFG Research Unit on Interactions of Essential Trace Elements in Healthy and Diseased Elderly, Potsdam-Berlin-Jena, Germany; c Department of Food Chemistry and Toxicology, Technische Universität Berlin, Berlin, Germany; d Department of Food Chemistry, Institute of Nutritional Science, University of Potsdam, Golm, Germany; e German Federal Institute for Risk Assessment (BfR), Berlin, Germany; f Max Rubner-Institut, Karlsruhe, 76131, Germany

**Keywords:** Copper, Zinc, Selenium, Mouse, Tissue, Trace element distribution

## Abstract

Trace elements such as copper, zinc, and selenium are essential micronutrients that play crucial roles in various physiological processes, mainly through their involvement in enzymes and regulatory proteins. A deficiency of any of these elements can impair physiological functions and lead to a range of symptoms. While copper deficiency is rare, e.g., vegans are particularly susceptible to inadequate intake of zinc and selenium. To investigate the effects of multiple simultaneous deficiencies, a feeding study was conducted in adult male and female C57BL/6Jrj mice receiving diets low in copper, zinc, and selenium. This approach enabled us to explore potential interactions between trace elements and to identify organ-specific effects based on their distribution profiles. We observed a substantial depletion of copper and selenium concentrations in the circulation and in almost all organs although to a varying extent. In contrast, zinc levels were well maintained and only declined in serum and bone. In line with the well-known antagonistic relationship between copper and zinc, our findings revealed that zinc deficiency mitigated symptoms of copper deficiency, which was most pronounced in female mice. Moreover, copper deficiency led to increased selenium concentrations in various organs, which, however, was not accompanied by generally higher selenoprotein expression. Therefore, it is essential to consider potential effects of single trace element deficiencies on other trace elements taking also combined effects into account.

## Highlights

• A parallel deficiency of zinc, copper, and selenium was established in adult mice.
• Males are more sensitive to copper deficiency than females.
• A zinc deficiency mitigates symptoms of copper deficiency.
• Low copper levels increase selenium concentrations in most organs tested.

## Graphical abstract

**Figure:**
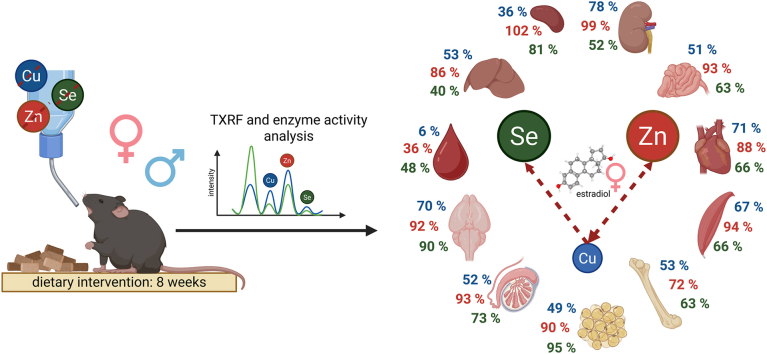

## 1 Introduction

Despite being required only in minute quantities, trace elements such as copper, zinc, and selenium are indispensable for maintaining cellular function, growth, and overall homeostasis. Their biological relevance arises largely from their incorporation into (metallo)enzymes and regulatory proteins that govern a wide range of critical processes including antioxidant defense, immune function, energy metabolism, and gene expression [[1], [2], [3]]. Copper serves as a cofactor for several key enzymes involved in respiration, neurotransmission or redox regulation [1]. These enzymes include cytochrome *c* oxidase, involved in cellular respiration in the mitochondrial electron transport chain, dopamine-β-hydroxylase, which synthesizes neurotransmitters, and superoxide dismutase 1 (Sod1), which catalyzes the dismutation of superoxide radicals to hydrogen peroxide, contributing to oxidative stress defense. One of the most prominent copper-dependent enzymes is ceruloplasmin (Cp), a ferroxidase primarily synthesized in the liver. Ceruloplasmin transports copper from the liver to peripheral organs. Additionally, the copper-dependent enzyme tyrosinase plays a crucial role in the initial steps of melanin production, which is responsible for skin, hair, and eye pigmentation in animals [4]. Zinc is structurally or catalytically required for over 300 enzymes and is involved in almost all aspects of cellular biology [5]. It stabilizes protein domains such as zinc fingers, enabling DNA binding to transcription factors, and regulates signaling pathways related to cell division, apoptosis, and immune response. Zinc also contributes to antioxidant defense through its role in Sod1 and is crucial for the activity of DNA polymerases and other enzymes involved in genome maintenance. Selenium's biological effects are primarily mediated through its incorporation into selenoproteins [6]. Among the most studied are glutathione peroxidases (Gpx), a family of enzymes that reduces hydrogen peroxide and lipid hydroperoxides to water or corresponding alcohols, thereby protecting cells from oxidative damage. Other essential selenoproteins include thioredoxin reductases (Txnrd) and iodothyronine deiodinases (Dio), which regulate cellular redox balance and thyroid hormone activity, respectively. A deficiency of one of these trace elements can result in a variety of symptoms, such as anaemia, neutropenia, and hair depigmentation for copper, skin lesions, impaired wound healing, compromised immunity, and loss of appetite for zinc, and thyroid dysfunction, muscle myopathy, and reproductive issues for selenium [7]. While dietary copper deficiency is relatively rare, genetic conditions can disrupt copper metabolism, leading to deficiency. Examples include Menkes disease, caused by mutations in the Atp7a gene, and aceruloplasminemia, caused by mutations in the Cp gene [8]. In comparison, zinc [9] and selenium [10] have either a low bioavailability or are inadequately supplied through food, especially in vegans [11,12]. For this reason, it is physiologically relevant not only to investigate the deficiency of a single trace element, but also how double or triple deficiencies manifest themselves.

Feeding a diet low in the trace element of interest is the gold standard for analyzing the consequences of their deficiency and assessing their physiological relevance. Respective mouse diets are commercially available. However, according to literature, such interventions must be carried out after weaning of the experimental animals or even during lactation in order to induce deficiency symptoms, which inevitably interfere with the animal's development [[13], [14], [15], [16], [17], [18], [19]]. Furthermore, deficiencies in multiple trace elements are rarely addressed simultaneously, and, to our knowledge, no mouse chow exists that allows for the intake of low concentrations of several trace elements. Accordingly, we started to address this issue by feeding weaned mice with a diet containing low but still detectable concentrations of copper, zinc, selenium, and iron. However, these studies primarily observed effects on the iron status, while copper, zinc, and selenium homeostasis was barely affected [20,21]. A new feed mixture revealed almost undetectable amounts of copper and zinc next to low amounts of selenium, which enabled us to modulate their status even in adult mice, resulting in fewer developmental effects. Accordingly, we can now address the question of how the distribution profiles of the trace elements copper, zinc, and selenium change under conditions of either sufficient or deficient supply. By analyzing parallel deficiencies, we aim to understand how these three trace elements are interconnected and how they influence the homeostasis of the others.

## 2 Material and methods

### 2.1 Animal experiment

Male and female C57BL/6Jrj mice (SOPF status, 9 weeks old, 8–9 mice per group) were obtained from Janvier Labs (Saint-Berthevin, France). All mice were acclimatized for two weeks in an environment maintained at 22 ± 2 °C and 55 ± 10 % humidity with a light/dark cycle of 14/10 h (dark phase from 8 p.m. to 6 a.m.). During these two weeks, animals received a commercially available chow diet (Altromin, Lage, Germany) that exceeded the nutritional requirements of mice for trace elements. Thereafter, all mice of the same sex were divided in a weight-matched manner into eight groups with varying trace element supply for copper, zinc, and selenium, with either one of these elements or a combination thereof being omitted. For this purpose, the animals were fed a semi-synthetic diet (Ssniff, Soest, Germany) which was deficient for copper, zinc, and selenium. In addition, the diet was also low in iron, manganese, and iodine (Table 1). But these three elements were not modulated in this experiment and accordingly were provided to all groups in sufficient concentrations via the drinking water (iron (II)chloride tetrahydrate (Sigma/Merck, Darmstadt, Germany), manganese (II)chloride tetrahydrate (Honeywell/Thermo Fisher Scientific, Darmstadt, Germany), potassium iodide (Honeywell)) throughout the experiment. In contrast, copper (copper (II)sulphate, Sigma), zinc (zinc sulphate pentahydrate, Sigma), and selenium (sodium selenite pentahydrate, Honeywell) were either supplied via the diet as the sole source and thus insufficiently, or were administered by adding them additionally to the drinking water in such a way that the final supply corresponded to the adequate amounts for mice (Table 1). Body weight as well as food and water consumption of all mice were constantly monitored during the intervention period. After 8 weeks, the mice were sacrificed, organs were removed, shock-frozen, and stored at −80 °C for analysis. Blood was collected by cardiac puncture and serum was obtained after complete coagulation at room temperature and 10 min of centrifugation (3000 g at 4 °C). All procedures were performed in accordance with the institutional guidelines of the Friedrich Schiller University of Jena/University Hospital and approved by the Thuringian State Authority for Consumer Protection (FSU-20-003).

**Table 1:** Trace element supply. Content of the trace elements copper, zinc, selenium, iron, manganese, and iodine in the semisynthetic diet, determined by ICP-MS/MS, compared to feeding recommendations [20]. ∗ below given detection limit; nba, not biologically available.

Element	Concentrations in the diet [mg/kg]	Feeding recommendations [mg/kg]
Copper	<0.0002∗	6.00
Zinc	<0.0009∗	30.0
Selenium	0.01	0.15
Iron	6.31	30.0
Manganese	1.20	10.0
Iodine	nba	0.15

### 2.2 Trace element concentrations

Prior to the experiment, feed samples were mechanically homogenized at room temperature. 20–50 mg of powdered feed samples were repeatedly subjected to acidic microwave digestion using HNO_3_ (65 % Suprapur, Merck) and H_2_O_2_ (30 %, Merck), followed by quantification using ICP-MS/MS (8800 ICP-QQQ-MS, Agilent Technologies, Waldbronn, Germany), as previously described in detail [22]. In addition, pieces of brain (cerebellum) were analyzed accordingly. Femurs were dried for 24 h at 120 °C, the dry weight was determined, and whole bones were dissolved in a mixture of ultrapure HNO_3_ and H_2_O_2_ (5:1, v/v) supplemented with germanium, iridium, and ^77^selenium (final concentration 10 μg/L) and rhodium (1 μg/L) as internal standard. An incubation at room temperature for 1 h, was followed by microwave-assisted digestion (MARS6, CEM, Kamp-Lintfort, Germany) at 1200 W, 20 min ramp, 20 min at 190 °C, yielding a clear solution prior to ICP-MS/MS analysis. The method was validated by using NIST 1486 bone meal certified reference material (Labmix24, Hamminkeln, Germany) demonstrating good recoveries for zinc (87.0 ± 2.8 %), selenium (94.7 ± 6.0 %), and copper (100.6 ± 14.6 %). The trace element content in brain and bone is given in mg per kg, normalized to wet and dry weight, respectively.

For all other organs and serum, concentrations of the elements copper, zinc, and selenium were examined using TXRF analysis (Bruker, Berlin, Germany), as previously described in detail [23]. Briefly, undiluted serum was mixed with an internal gallium standard (1 mg/mL, Alfa Aesar/Thermo Fisher Scientific) and used directly for quantification. Organs were pulverized under liquid nitrogen and suspended in RIPA buffer (radioimmunoprecipitation assay, 50 mM Tris (Applichem, Darmstadt, Germany), 150 mM NaCl (Carl Roth, Karlsruhe, Germany), 2 mM EDTA (Applichem), 0.5 % sodium deoxycholate (Merck), 0.1 % sodium dodecyl sulphate (Applichem), 1 % NP-40 (Merck/Millipore, Burlington, MA, USA) with 1 mg/mL protease inhibitor (Merck/Millipore)). The organ lysates were mixed with 1 mg/L yttrium (Merck) as an internal standard. The concentration of trace elements in serum was expressed in μg per liter, while the concentration in organs was normalized to protein content and expressed in μg per gram of protein.

### 2.3 Free zinc and copper concentrations

Free zinc (fZn) was determined as described before [24] using the low molecular weight fluorescent probe Zinpyr-1. The determination of free copper (fCu) was based on the use of the Cu^2+^-binding fluorescein peptide sensor (FP4). The procedure is similar to that described in Ref. [25] for humans and rats, except that the initial incubation with the peptide was carried out for 90 min instead of 60 min.

### 2.4 Enzyme activities

The serum Gpx activity was determined in a NADPH-consuming glutathione reductase-coupled assay, which has been described in detail before [26] using H_2_O_2_ as substrate. To measure the oxidase activity of copper-dependent Cp in serum, the conversion of O-dianisidine chloride is considered, which has been described elsewhere [27].

For the analysis of hepatic Dio1 activity, frozen liver samples were pulverized, mixed with homogenization buffer (250 mM d-sucrose, 20 mM Hepes, 1 mM EDTA, pH 7.4) and underwent subsequent sonication (2 × 10 pulses, UP50H, Hielscher, Teltow, Germany). Homogenates were adjusted to a uniform concentration, and a total of 40 μg protein was added to each reaction. Relative Dio1 activity was determined with the Sandell-Kolthoff-reaction as previously described [28]. An iodide standard curve was used to interpolate the absolute Dio1 activity in pmol/mg∗min.

### 2.5 mRNA expression

Total RNA was isolated from liver samples according to the manufacturer's instructions for the Trizol reagent (Invitrogen, Thermo Fisher Scientific). Of this, 10 μg of RNA was subjected to DNase I digestion (2U; Thermo Fisher Scientific), followed by reverse transcription in a final volume of 40 μL (qScript cDNA synthesis, Quanta BioSciences/VWR, Darmstadt, Germany). For amplification of the cDNA, 1x PerfeCTa SYBR Green Supermix (Quanta BioSciences), cDNA and 250 nM oligonucleotides (sequences are listed in Table 2) were mixed in a total volume of 10 μl and subjected to a thermocycling program consisting of 3 min at 95 °C, followed by 40 cycles of 15 s at 95 °C, 20 s at 60 °C and 30 s at 72 °C. Quantitative real-time polymerase chain reaction (qRT-PCR) was performed using the Mx3005 P real-time PCR system (Stratagene, Agilent Technologies). Copy numbers were calculated using standard curves. Expression levels were normalized using a composition factor based on the reference genes hypoxanthine phosphoribosyltransferase 1 (Hprt), ribosomal protein L13a (Rpl13a), and ribosomal protein L37 (Rpl37).

**Table 2:** Oligonucleotide sequences (5’→3’).

Gene	RefSeq-ID	Sequence
Dio1, deiodinase 1	NM_007860.3	GGGATTTCATTCAAGGCAGCAGG
		TGTGGAGGCAAAGTCATCTACGA
Gpx1, glutathione peroxidase 1	NM_008160.5	GAAGAGATTCTGAATTCCCTCAA
		GAACTTCTCAAAGTTCCAGGCA
Gpx4, glutathione peroxidase 4	NM_001037741.2	GCTGGGAAATGCCATCAAATGGA
		ACGGCAGGTCCTTCTCTATCAC
Hprt 1, hypoxanthine guanine phosphoribosyl transferase 1	NM_013556.2	GCAGTCCCAGCGTCGTG
		GGCCTCCCATCTCCTTCAT
Rpl13a, ribosomal protein L13a	NM_009438.5	GTTCGGCTGAAGCCTACCAG
		TTCCGTAACCTCAAGATCTGCT
Rpl37, ribosomal protein L37	NM_026069.3	GCAACAAGACGCACACGCTG
		GAATCCATGTCTGAATCTGCGGT
Selenop, selenoprotein P	NM_001042613.1	CTCATCTATGACAGATGTGGCCGT
		AAGACTCGTGAGATTGCAGTTTCC
Selenow, selenoprotein W	NM_009156.2	ATGCCTGGACATTTGTGGCGA
		GCAGCTTTGATGGCGGTCAC
Txnrd1, thioredoxin reductase 1	NM_001042523.1	AGCAGCTAAGGAGGCAGCCA
		TTTCCAGCCATAGTTGCGCGAG

### 2.6 Statistical analysis

Statistical evaluation was performed in GraphPad Prism 9 (GraphPad Software, La Jolla, USA). After removal of statistical outliers by Grubbs test, normal distribution (Shapiro-Wilk test) and variance (Levene test) were tested. Data are presented as mean of experimental animals. Differences between groups were analyzed using one-way (testis) or two-way ANOVA with Bonferroni's post-hoc test. The correlation coefficient was calculated according to Spearman (denoted as rS). P < 0.05 was considered statistically significant.

## 3 Results

### 3.1 Characteristics of the animals were modulated by copper and zinc deficiency and their interdependence

**Fig. 1:**
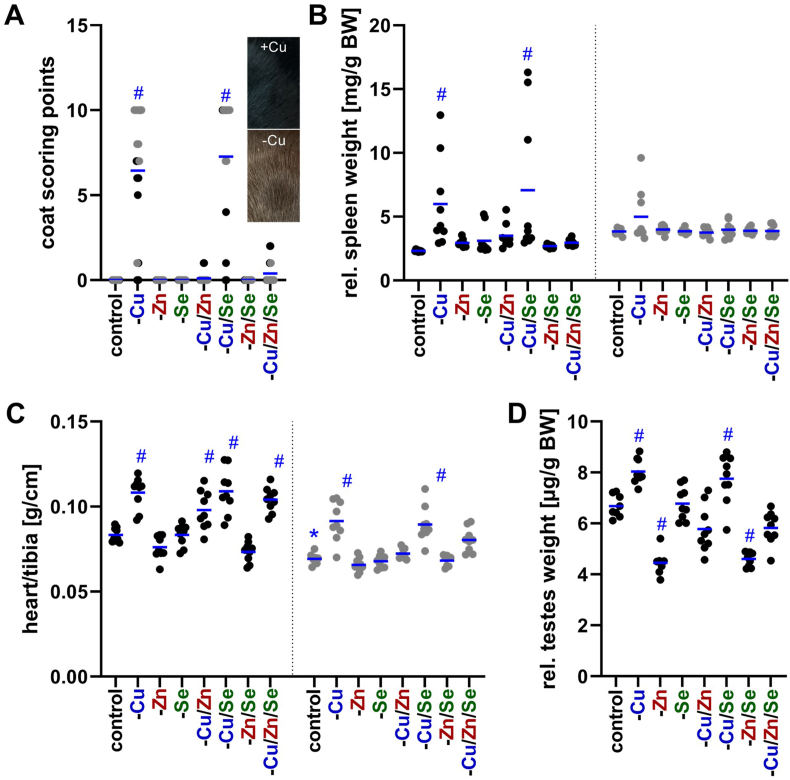
**Characteristics of the C57BL/6Jrj mice after eight weeks of dietary intervention with insufficient copper, zinc, and selenium supply, as well as combinations thereof.** Group-dependent analysis of change in coat color from black to brown (A), and organ weights of spleen (B), heart (C), and testes (D). The organ weights of the spleen and testes were normalized to the body weight (BW) of the animals at the time of sacrifice, while the heart weight was normalized to tibia length, which did not vary between groups. The individual measurements for each mouse and their average values are shown. The data for the males are displayed in black and the data for the females in grey. Statistical testing based on Two-Way ANOVA (A-C) or One-Way ANOVA (D) and Bonferroni's post-test with ^#^p < 0.05 compared to sex-specific control and ∗p < 0.05 compared to male control (only shown for adequately fed animals).

The first visible effect of an eight-week dietary intervention with varying supply of the trace elements copper, zinc, and selenium was a change in coat color turning the original black coat of the C57BL/6Jrj mice into brown in some cases, which has been described before as a sign of copper deficiency [4]. We used a scoring system for each mouse to quantify when and to what extent the color shift took place. Clearly, the animals of the -Cu or -Cu/Se group received the highest scores, irrespective of sex (Fig. 1A). When copper deficiency was combined with zinc deficiency (-Cu/Zn and -Cu/Zn/Se groups), there was virtually no detectable effect on coat color, indicating a rescue of copper deficiency by a combined zinc deficiency. The development of the body weight of the mice did not mirror this effect. In this case only adequately supplied animals and -Se animals gained weight (127.0 ± 6.8 % and 116.8 ± 10.4 %, respectively), while the remaining groups showed almost no weight gain during the eight weeks (Fig. S1A), indicating some metabolic impairments due to both the deficiency of Cu and Zn.

Next, organ weights were quantified in relation to the trace element supply. While the liver showed no weight differences between groups when normalized to body weight (Fig. S1B), group-specific changes in relative spleen, heart, and testes weight were observed. For spleen an increased relative weight was detected in -Cu and -Cu/Se males but not in the groups in which copper and zinc deficiencies were combined (Fig. 1B). Thus, as described for the coat color change, a combined zinc deficiency was able to rescue the -Cu effect in male mice. However, in females, only a few -Cu animals showed a mild splenomegaly indicating an overall milder effect than in males. Regarding changes in heart weight, males were again more susceptible than females showing an increased heart weight in all four groups with copper deficiency (Fig. 1C). However, females were only affected in the -Cu and -Cu/Se groups again indicating some improvement by a combined zinc deficiency. Overall, the heart weight of females was always lower than that of males. Regarding the testes weight, zinc and copper deficiencies had opposite effects (Fig. 1D). While zinc deficiency (-Zn and -Zn/Se) was associated with a decrease in relative testes weight, copper deficiency (-Cu and -Cu/Se) led to an increase in testes weight. In case of a combined copper and zinc deficiency (-Cu/Zn and -Cu/Zn/Se) there was no significant difference in testes weight but only a minimal non-significant reduction. Selenium deficiency did not show any effect on testes size. Overall, signs of copper deficiency including browning of coat, increased relative spleen and heart weight could be rescued by zinc deficiency, although this was partially sex dependent.

### 3.2 Serum analysis revealed clear deficiencies of all three trace elements

**Fig. 2:**
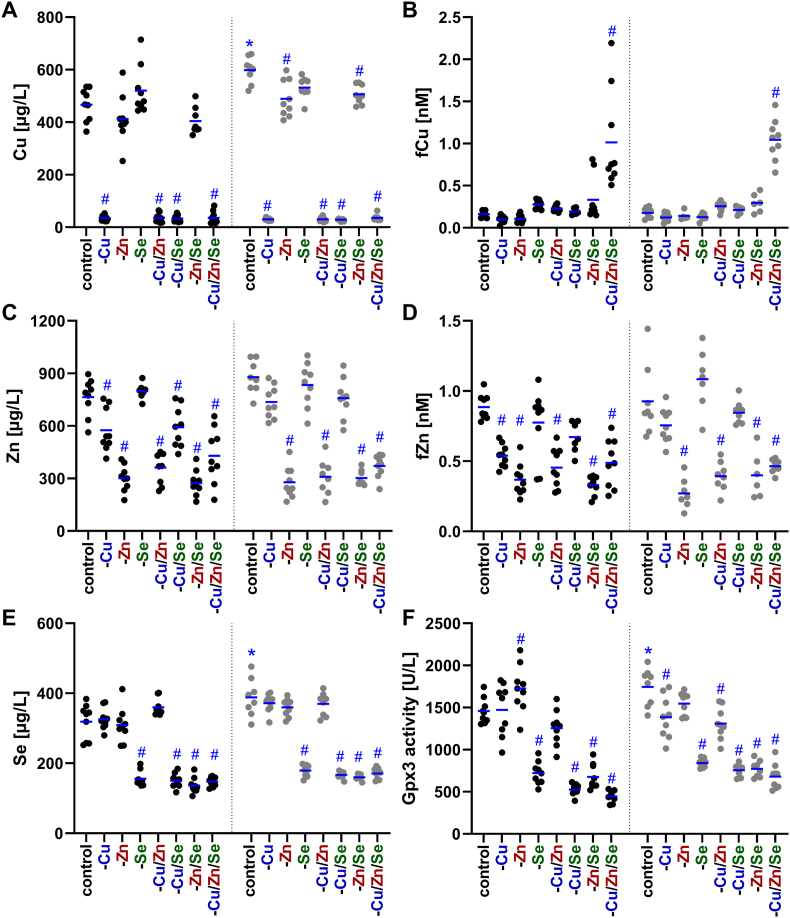
**Changes of trace element profiles and biomarkers in serum of C57BL/6Jrj mice.** Concentrations of copper (Cu; A), zinc (Zn; C), and selenium (Se; E) were analyzed in the serum of 19 weeks old male and female C57BL/6Jrj mice (n = 8–9) receiving a diet with varying trace element supply. Serum concentrations were determined using TXRF. Biomarkers were detected by fluorescent probes to assess free copper (fCu; B) and free zinc (fZn; D) in serum. As selenium biomarker, Gpx3 activity (F) was analyzed by a NADPH-consuming glutathione reductase coupled assay. The individual measurements for each mouse and their mean values are shown. The data for the males are displayed in black and the data for the females in grey. Statistical testing based on Two-Way ANOVA and Bonferroni's post-test with ^#^p < 0.05 compared to sex-specific control and ∗p < 0.05 compared to male control (only shown for adequately fed animals).

Next, we aimed to characterize whether the reduced supply of trace elements for 8 weeks was able to modulate serum concentrations of the trace elements and related biomarkers (Fig. 2). Copper and zinc in the feed were below the detection limit, indicating a drastic reduction of supply (6 and 30 ppm, respectively, are considered as adequate supply). The serum concentration of copper was substantially reduced to 16 %, regardless of the combination of trace elements and sex, when no copper was administered (Fig. 2A). In the groups with adequate copper intake, female animals always had about 18 % higher serum copper concentrations than males. A low zinc supply (-Zn, -Zn/Se) reduced serum copper concentrations under conditions of adequate copper intake. In females, the reduction was on average 17 %, while in males this was only a trend (reduction by approximately 12 %). The measurement of copper-dependent enzyme activity of Cp correlated very well with the copper concentration in serum (rS = 0.871, p < 0.001) and behaved equivalently (Fig. S2). Free copper II ions, in contrast, showed almost no correlation with serum copper concentrations (rS = −0.192, p = 0.03) and was not downregulated in copper deficient groups (Fig. 2B). Minimal increases were observed in cases of two combined trace element deficiencies (-Cu/Zn, -Cu/Se, -Zn/Se). However, clear differences compared to the adequately supplied control animals were evident in the group with a combined trace element deficiency of copper, zinc, and selenium (-Cu/Zn/Se). Here, the mean fCu concentration was more than five times higher than in the corresponding control animals. This might indicate that the triple deficiency was harmful for one or several organs resulting in leakage of fCu.

Similar to copper, the zinc supply was strongly reduced in the -Zn groups resulting in serum concentrations of about 40 % of adequate concentrations (Fig. 2C). However, zinc concentrations did not decrease only as a result of insufficient zinc intake, but also due to a deficiency of copper (either -Cu or -Cu/Se), which was accompanied by a reduction of 20 % only significant in males. In contrast to fCu, the concentration of free zinc displayed the same pattern as the total zinc concentration (Fig. 2D), which is also reflected by a correlation of rS = 0.888 (p < 0.001).

In contrast to the interrelationship of zinc and copper, the serum selenium concentration was only dependent on the selenium supply (Fig. 2E). The selenium concentration was reduced by 55 % in all selenium-deficient groups. As described for copper, females generally showed higher basal levels than males. The activity of the selenium-dependent enzyme Gpx3 also showed a strong selenium dependency (Fig. 2F), with an average decrease in activity of 54 % when selenium was lacking. In contrast to selenium concentrations, Gpx3 activity was increased by 18 % in males of the -Zn group, while in females a dependence on copper was evident, with Gpx3 activity being 23 % lower on average in -Cu and -Cu/Zn-deficient animals. Overall, the deficiency of all three trace elements could be confirmed by analyzing their serum concentrations. Interactions between copper and zinc have been observed, with a deficiency of one reducing the serum concentration of the other.

### 3.3 Hepatic concentrations of copper and selenium were clearly reduced while zinc was only mildly affected in females and well maintained in males

**Fig. 3:**
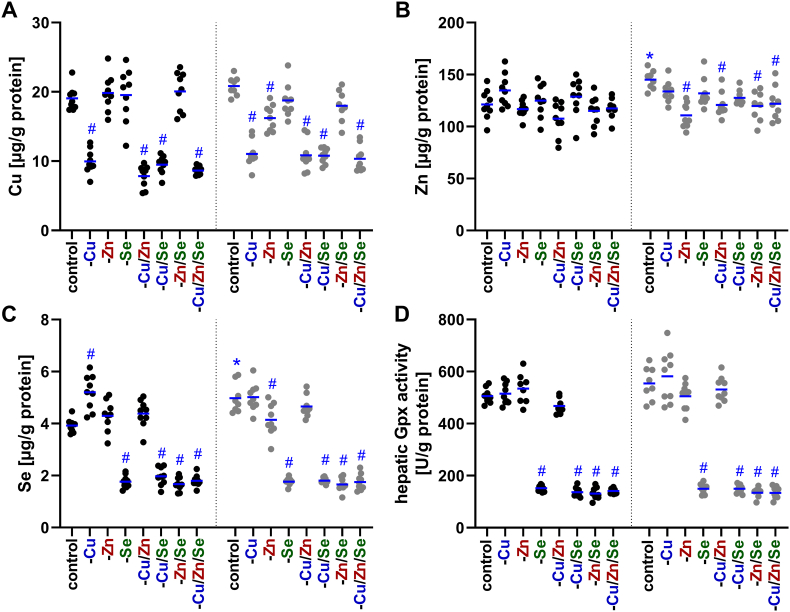
**Alterations of trace element profiles in liver of C57BL/6Jrj mice.** Concentrations of copper (Cu; A), zinc (Zn; B), and selenium (Se; C) were analyzed in liver tissue of 19-weeks-old male and female C57BL/6Jrj mice (n = 8–9) receiving a diet with varying levels of these trace elements. Hepatic concentrations were determined using TXRF and normalized to protein content. The selenium status was additionally validated by GPX activity (D), based on an NADPH-consuming glutathione reductase coupled assay and normalized to protein content. The individual measurements for each mouse and their mean values are shown. The data for the males are displayed in black and the data for the females in grey. Statistical testing based on Two-Way ANOVA and Bonferroni's post-test with ^#^p < 0.05 compared to sex-specific control and ∗p < 0.05 compared to male control (only shown for adequately fed animals).

In line with very low serum concentrations, hepatic copper concentrations decreased by about 50 % after the dietary intervention in all copper deficient groups (Fig. 3A). In female animals, a low zinc supply also resulted in a 22 % reduction in hepatic copper levels fitting to reduced hepatic zinc levels observed in these groups but not in males (Fig. 3B). The decline of hepatic zinc concentrations was on average 18 % in all four zinc-deficient groups while in males hepatic zinc levels were well maintained independent of the trace element supply. This indicates that only in females a low zinc intake resulted in reduced hepatic zinc concentrations and accordingly only in females an interaction of copper and zinc was detectable.

In comparison, hepatic selenium concentrations were clearly dependent on selenium intake (Fig. 3C) with a decline of 65 % in females and 54 % in males. This sex difference arose from a 27 % higher selenium concentration in the liver of female animals compared to male animals. Hepatic Gpx activity also revealed a clear downregulation upon selenium deficiency to 27 % regardless of the trace element combination (Fig. 3D). However, in response to isolated copper deficiency an increase in selenium concentrations was only observed in males which was not reflected by an increase in hepatic Gpx activity. Apart from this interaction, lower copper and selenium concentrations were found in females of the -Zn group (Fig. 3A–C) indicating a sex-specific interaction.

### 3.4 Copper was strongly depleted across all organs analyzed

The investigation of the effects of varying trace element intake on extra-hepatic organs revealed that inadequate copper intake affected all organs examined. In the spleen, the decline of the copper content was particularly pronounced in both sexes (Table 3), whereby the size of the organ was primarily increased in the males and especially in -Cu animals (Fig. 1B). Animals of the -Cu/Zn/Se group showed no alteration in organ size but exhibited the same copper content as the other copper-depleted groups, which was reduced by around 63 % compared to the control. Subcutaneous fat and testes were also strongly affected by copper deficiency, showing a reduction to 50 % of the copper content compared to the control animals. The copper content in the intestine and femur was reduced by around 45 % with insufficient copper supply. The smallest decrease in copper content in the copper-depleted groups compared to the controls was observed in the kidneys, heart, brain, and muscle. Here the copper concentration fell between 17 % (kidney) and 33 % (muscle). Interestingly, there was a slight increase in copper concentrations in the femur of males in connection with -Se, whereas in females an increase was more apparent in connection with -Zn and -Zn/Se.

**Table 3:**
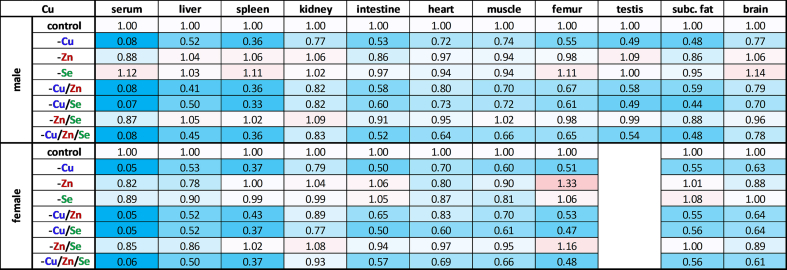
Relative copper distribution across murine organs. Relative total copper content in serum and various organs of 19-weeks-old male and female C57BL/6Jrj mice (n = 8–9) receiving a diet with varying levels of the trace elements copper, zinc, and selenium. The trace element concentrations were normalized (except for serum) to either protein content, wet weight (brain) or whole bone dry weight (femur). Trace element concentrations were expressed as fold change compared to controls for each sex. Blue indicates a downregulation and red an upregulation in comparison to control.

### 3.5 Zinc concentrations remained largely stable, except for femurs

Upon closer examination of the different organs with respect to zinc content, no drastic changes became apparent following zinc depletion (Table 4). The most pronounced changes were observed in the femur, where the zinc content in the zinc-deficient groups dropped by around 19 % in males, while a reduction of 35 % was observed in females. Thus, zinc could be kept very stable in the body despite insufficient intake. Regarding trace element interactions, a small increase in zinc was observed in the spleen depending on the copper supply in males but this appears to be connected to the enlargement of the organ.

**Table 4:**
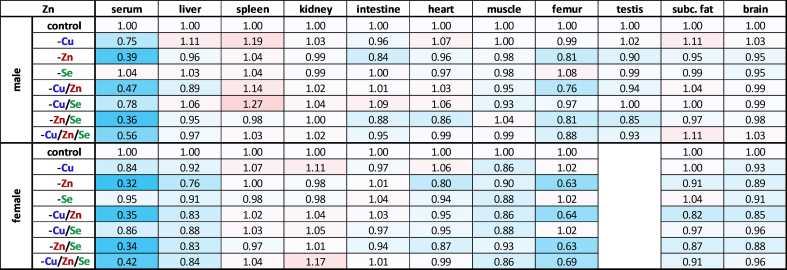
Relative zinc distribution across murine organs. Relative total zinc content in serum and various organs of 19-weeks-old male and female C57BL/6Jrj mice (n = 8–9) receiving a diet with varying levels of the trace elements copper, zinc, and selenium. The trace element concentrations were normalized (except for serum) to either protein content, wet weight (brain) or whole bone dry weight (femur). Trace element concentrations were expressed as fold change compared to controls for each sex. Blue indicates a downregulation and red an upregulation in comparison to control.

### 3.6 Selenium concentrations were declining following the established organ hierarchy but were increased upon copper deficiency

The selenium content of most organs, again, was strongly influenced by external intake (Table 5). Next to the liver, the selenium content in the kidneys (−44 %) decreased the most when intake was insufficient. In the intestines, subcutaneous fat, and muscle, selenium concentrations fell by an average of 31 %, while in the heart and femur they were reduced by 27 %. The spleen appears to be less dependent on external selenium intake, with a 15 % reduction in selenium concentration. In testes and brain, selenium concentrations fell by only 8 %, after an eight-week period on a selenium-deficient diet. It is worth noting that the decrease in selenium content was slightly less pronounced in spleen and femur when selenium deficiency occurred in combination with zinc deficiency (-Zn/Se, -Cu/Zn/Se). However, zinc deficiency alone had no significant effect on selenium concentrations. In contrast, insufficient copper intake (-Cu, -Cu/Zn) was associated with an increased selenium concentration (+12–33 %), particularly in the femur, kidney, muscle, and heart fitting to the effect observed in the male liver (+22 %). In spleen and subcutaneous fat, this correlation was only tentative.

**Table 5:**
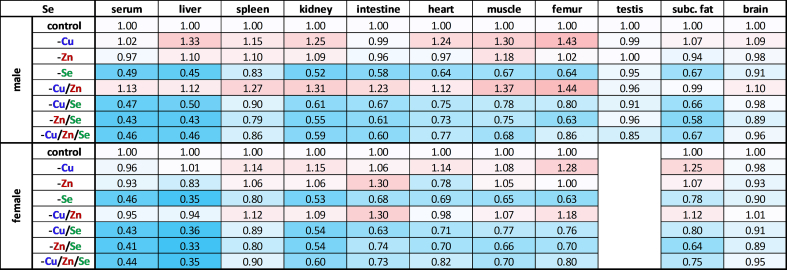
Relative selenium distribution across murine organs. Relative total selenium content in serum and various organs of 19-weeks-old male and female C57BL/6Jrj mice (n = 8–9) receiving a diet with varying levels of the trace elements copper, zinc, and selenium. The trace element concentrations were normalized (except for serum) to either protein content, wet weight (brain) or whole bone dry weight (femur). Trace element concentrations were expressed as fold change compared to controls for each sex. Blue indicates a downregulation and red an upregulation in comparison to control.

### 3.7 Selenoprotein expression in the liver in response to copper deficiency

**Fig. 4:**
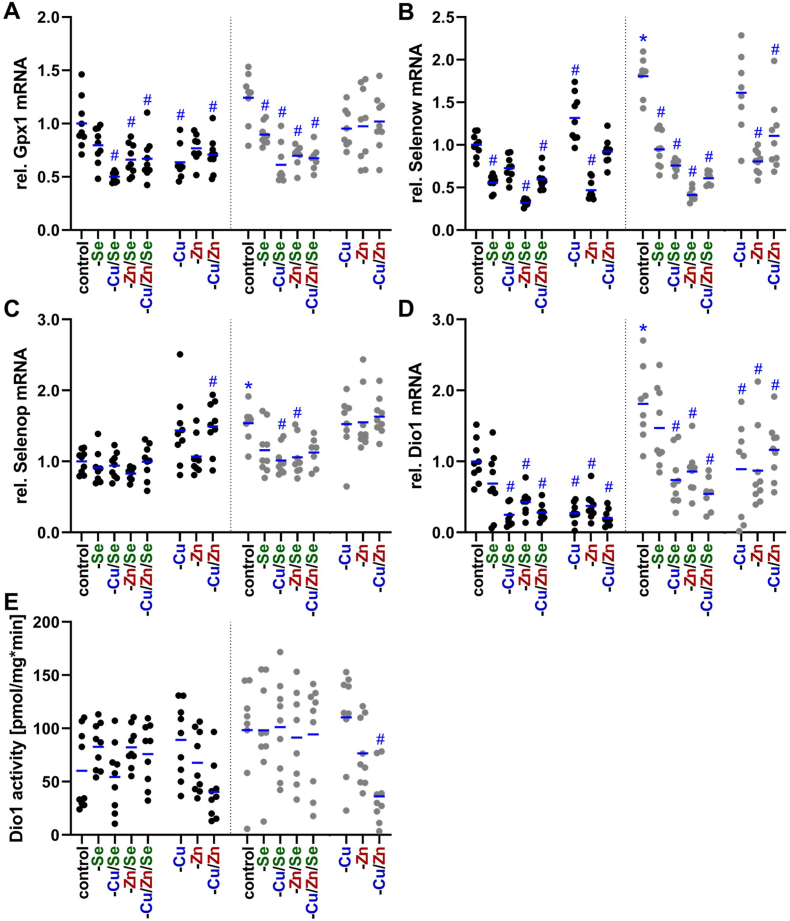
**Relative mRNA expression of selenoproteins in liver of C57BL/6Jrj mice and hepatic deiodinase activity.** Relative expression levels of selenoproteins like glutathione peroxidase 1 (Gpx1; A), selenoprotein W (Selenow; B), selenoprotein P (Selenop; C), and deiodinase 1 (Dio1, D) were determined by qRT-PCR in liver tissue of 19-weeks-old male and female C57BL/6Jrj mice (n = 8–9) receiving a diet with varying levels of the trace elements copper, zinc, and selenium. Expression levels were normalized to a composition factor based on Hprt, Rpl13a, and Rpl37. All results are expressed as fold change compared to male controls. Hepatic Dio1 activity (E) was determined by Sandell-Kolthoff-based activity assays. Data for the males are displayed in black and for the females in grey. Statistical testing based on Two-Way ANOVA and Bonferroni's post-test with ^#^p < 0.05 compared to sex-specific control and ∗p < 0.05 compared to male control (only shown for adequately fed animals).

As several organs showed an upregulation of selenium in response to copper deficiency, we aimed to further characterize the mRNA expression of selenoproteins in the liver. The two selenium-sensitive selenoproteins Gpx1 (Fig. 4A) and Selenow (Fig. 4B) were downregulated in all selenium-deficient groups (though sometimes only showing a trend in males) as expected while other selenoproteins such as Gpx4 (Fig. S3A) and Txnrd1 (Fig. S3B) were completely unaffected by any of the trace element deficiencies. Selenop (Fig. 4C) and Dio1 (Fig. 4D) which rank in the middle of the selenoprotein hierarchy showed a moderate response to selenium deficiency being more pronounced in females than in males. Considering the effect of copper deficiency we observed a mixed response. In line with the selenium concentration of male mice, mRNA levels of Selenop and Selenow were upregulated during copper deficiency. However, Gpx1 and Dio1 showed the opposite being downregulated upon copper deficiency, which was observed in all -Cu groups. Interestingly, zinc deficiency modulated selenoprotein expression and reduced Selenow and Dio1 expression to an extent comparable to selenium deficiency. Enzymatic activity of Dio1 in the liver was rather heterogenous within groups (Fig. 4E). Nevertheless, a decreased Dio1 activity was present in the female -Cu/Zn group. Thus, besides selenium also copper and zinc deficiency modulated hepatic selenoprotein expression.

## 4 Discussion

Trace elements are crucial for various body functions including development and growth [18,19,29]. However, inducing trace element deficiencies (like zinc and especially copper) in animal studies is commonly done through early dietary interventions, usually starting shortly after weaning or even prenatally [[13], [14], [15]]. Studying effects of deficiencies during such critical periods gives insights into consequences of early-life deficiencies, which may be irreversible [30], but limit the comparability to effects of deficiencies occurring later in life. Accordingly, we established a feeding model that can be used for the parallel restriction of the nutritional supply of copper, selenium, and zinc in adult mice.

Surprisingly, this study revealed a pronounced copper deficiency that affected all organs, despite the intervention commencing in adult mice rather than after birth. It appears that copper is mobilized very quickly when the supply is insufficient (unpublished data), leading to a pronounced decrease in copper concentrations, particularly in serum, liver, spleen, intestine, femur, testes, and adipose tissue (Table 3). These results fit to the established copper hierarchy, which assumes that copper loss in the heart and brain is significantly delayed [31]. After nine weeks of copper restriction, the copper levels of heart and brain were only reduced by 3 % and 1 %, respectively [31]. This indicates substantially lower copper mobilization than in our study. But in other studies, e.g., in male Hsd:ICR (CD-1) outbred albino mice a 85 %, 75 %, and 35 % decrease in copper levels of the liver, brain, and kidney was shown, respectively [13]. Overall, we provide evidence for an efficient mobilization of copper in adult organisms, especially from the liver. In line with this, an inadequate selenium intake decreased selenium concentrations mostly in the liver, kidney, and serum, while testes and brain were largely resistant to limited selenium intake (Table 5). These results are consistent with the predominant organ hierarchy of selenium, primarily controlled by Lrp8 [32]. Lrp8 plays a pivotal role in facilitating the uptake of selenium as SelenoP in brain and testes, thus ensuring its utilization for maintaining the selenoprotein expression of these two organs [33]. In contrast to copper and selenium, the insufficient supply of zinc did not lead to a uniform loss in the organs, despite undetectable amounts in the feed. Instead, zinc concentrations remained constant in all examined organs, except for serum (−60 %), femur (−27 %, with greater reductions in female animals), and liver (−18 %, only observed in females) (Fig. 3B; Table 4). In the literature, feeding a less severe zinc deficiency than in this study for 3–4 weeks reduced zinc concentrations in the serum, muscles, and bones [34,35], while others observed decreases in zinc concentrations in serum, liver, lung, and heart [36]. Due to these inconsistencies, it is discussed that the body's mobilization of zinc from its storage organs (i.e., bones and muscles) is gradual and that the turnover of zinc-containing proteins may be substantially diminished in response to zinc deficiency. However, it must be acknowledged that analyzing whole organs often fails to account for potential local variations in concentrations. Furthermore, the potential for contamination with zinc from the animals' housing material remains a possibility, although this was mitigated to the greatest extent possible through the utilization of non-consumable variants during the trial. Accordingly, it needs to be considered that in this study zinc concentrations were well maintained in most organs while a functional decline of copper- and selenium-dependent enzymes was detectable.

The main aim of this study was the analysis of potential interactions of trace elements under conditions of single or combined deficiencies. In a previous study of combined deficiency of several trace elements (iron, zinc, copper, iodine, and selenium), albeit only moderate for 9 weeks, we already observed physical effects such as lower weight gain, splenomegaly, and cardiomegaly when the intervention started at weaning of the animals [20]. The combined and isolated deficiencies show that both low copper and especially low zinc supply are decisive for weight gain (Fig. S1A), as previously reported, at least for zinc in mice [37] and rats [38]. Both deficiencies are also associated with changes of the coat color. While zinc deficiency is associated with brittle hair and loss of hair [17], copper deficiency is associated with depigmentation (silver-grey or lighter), as the tyrosinase involved in melanin production is copper-dependent [4]. While brittle or even the loss of hair was not observed, copper deficiency was associated with a change in coat color from the 5th week of intervention (Fig. 1A). However, when the copper and zinc supply were reduced simultaneously, this depigmentation did not occur until the last week of the intervention, which suggests that a simultaneous zinc deficiency delayed effects of copper deficiency. In line with this, an enlargement of the spleen and heart was observed under copper deficiency but not in groups with combined copper and zinc deficiency (Fig. 1). In the literature, copper is associated with both a reduction [39] and enlargement of the spleen [29], whereas zinc deficiency is associated with splenic fibrosis [40]. Also, it is well documented that nutritional copper deficiency induces cardiac hypertrophy in rodents [41], which is characterized by an enlargement and a higher weight of the heart, probably based on altered gene expression in cardiomyocytes inducing a myocardial remodeling program [42]. Thus, we could clearly show that especially in females a combined zinc deficiency can counteract the effects of copper deficiency. This is very surprising because no overt zinc deficiency could be detected when analyzing total zinc concentrations, but obviously counter regulation or adaptive processes took place affecting copper concentrations and downstream copper-dependent processes even though zinc levels were still maintained. Obviously, these processes did not involve serum fCu as this was the only parameter that was only increased upon a combined deficiency of copper, selenium, and zinc but not under any other feeding condition (Fig. 2B).

Interactions between copper and zinc are well established in the literature [43] and are based on similar chemical and physical properties of copper and zinc ions, which compete for similar binding sites, e.g., in metallothionein. High zinc intake can impair copper absorption by inducing metallothionein in the intestine, which binds and sequesters copper, leading to its excretion through shedding of intestinal cells [44]. Conversely, high copper levels can also affect zinc metabolism, although the effect seems to be less consistent [43]. Accordingly, copper-zinc ratios are very helpful when considering these interactions. Based on the varying severity of copper and zinc deficiency in the different organs, copper-zinc ratios exhibited variations (Table S1). In serum, the substantial decline in copper in relation to zinc lead to the lowest copper-zinc ratio in the copper-depleted animals, while the ratio was highest in the sera of the -Zn and -Zn/Se animals. These changes could be attributed to an interaction at the level of intestinal cells. In -Cu/+Zn conditions, the presence of zinc induces metallothionein synthesis, resulting in the accumulation of copper within intestinal cells [44]. Consequently, even less copper could enter the bloodstream and exacerbate the deficiency, which may account for its early manifestation e.g., by coat changes. The decrease of zinc in the serum manifested more slowly and could also be self-reinforcing due to the simultaneous presence of copper in the -Zn and -Zn/Se animals, which is why lower mean zinc levels were recorded here than in -Zn/Cu (Table 4). Additionally, a decrease in zinc levels was not only evident in the case of zinc deficiency, but also in copper deficiency (-Cu, -Cu/Se) (Fig. 2C). This suggests possible transcriptional (e.g., via the metal regulatory transcription factor 1), translational, and molecular interactions of copper and zinc at the level of transport (e.g., Cp), storage (e.g., metallothionein) or cellular processes such as oxidative stress as both elements are important for Sod1 activity.

In addition to serum, an elevated copper-zinc ratio was observed in the femur of female -Zn and -Zn/Se mice (Table S1). This finding suggests that females exhibit a markedly superior zinc mobilization from the bones which appears also to be the case for the liver (Fig. 3B). Estrogen has been shown to impede the activity of osteoclasts under physiological conditions, thereby mitigating bone resorption [45]. Zinc is also associated with a healthy bone matrix, as it is thought to be involved in the synthesis of the collagen matrix, mineralization, and bone turnover [46]. In line with zinc, also the calcium and phosphate concentrations in the bones of female mice were reduced only in the -Zn group (Fig. S4) indicating an enhanced bone turnover. In particular, the direct link between zinc and estrogens is likely to be crucial for bone health, as zinc acts as a cofactor in numerous hormone-producing enzymes (e.g., aromatase, 3- and 17-beta-hydroxysteoride dehydrogenase) and estrogen receptors have been found to contain zinc finger domains. Thus, zinc deficiency is associated with a reduction in estrogen [47,48]. In addition, estrogen affects zinc homeostasis by modulating the expression of zinc transporters (ZIPs and ZnTs), thereby influencing its distribution and mobilization [49]. This may explain why zinc is mobilized differently in females than in males.

Next to copper and zinc, an interaction between copper and selenium was also identified. Copper deficiency increased selenium concentrations in many organs, particularly in the femur which was more pronounced in males than in females (Table 5). Such an interaction between copper and selenium has been described in cases of copper excess in HepG2 cells and in Wilson's disease models, which are associated with copper accumulation in the liver [27,50]. Thus, excess copper disrupts Selenop transport through the late Golgi and, consequently, the secretion of Selenop from the liver. This can have a secondary effect on the expression and activity of selenoproteins, such as Gpx1 resulting in lower Gpx activity. In the Wilson mouse model (20 weeks old), decreased selenium concentrations were observed in the liver, brain, and heart with increasing copper concentrations, though the mRNA levels of selenoproteins remained largely preserved but selenoprotein synthesis was reduced [51]. Furthermore, increased copper intake (42 mg Cu/kg diet) with low (0.03 mg Se/kg diet) or normal (0.05 mg Se/kg diet) selenium intake was associated with decreased intestinal selenium absorption and increased renal excretion [52]. Additionally, increased copper intake was associated with elevated selenium levels in the liver and kidneys, whereas altered selenium intake did not affect copper levels. The observed correlation between copper deficiency and increased selenium concentrations could be due to impaired copper-dependent enzymes. Inadequate enzyme function, such as that of Sod1, might lead to increased oxidative stress. This may result in the upregulation of antioxidant proteins, such as selenoproteins, e.g., glutathione peroxidases and thioredoxin reductases, which is associated with an increased requirement for selenium. However, this hypothesis is contradicted by the finding that increased hepatic selenium content in -Cu males resulted in reduced Gpx1 mRNA expression (Fig. 4A) and was not reflected by altered Gpx activity (Fig. 3D). Additionally, significant changes in hepatic Dio1 mRNA expression (Fig. 4D) were mostly not accompanied by changes in Dio1 activity (Fig. 4 E), again suggesting poor translation of expression levels to protein activities in selenoproteins [53]. Only the female -Cu/Zn group, showed a parallel reduction in Dio1 transcription and its enzymatic activity. The fact, that there were sex-dependent differences in selenium accumulation in response to copper deficiency, as well as higher selenium levels in serum, liver, and kidneys of female animals compared to males (Table 5), could again be based on estrogen dependence. In fact, it has already been shown in ovariectomized rats that estrogen administration modulates the uptake and distribution of selenium in tissues such as blood and liver [54].

In summary, our results show that interactions among trace elements occur not only when there is an excess of trace elements, which can be more easily induced in cell cultures and animal models, but also when there is a deficiency. In this case, one deficiency can partly cancel out another, as shown for copper and zinc. The exact molecular mechanisms remain unclear and require further analysis. But the study shows very clearly that even in the case of single trace element deficiencies, possible effects on other trace elements must be considered. This is of major importance for humans who, for example, reduce their meat intake and consequently limit the amount of selenium and zinc supplied through their diet in parallel.

## CRediT authorship contribution statement

**Kristina Lossow:** Conceptualization, Data curation, Formal analysis, Investigation, Methodology, Validation, Visualization, Writing – original draft, Writing – review & editing. **Maria Maares:** Formal analysis, Investigation, Validation, Writing – review & editing. **Tom Heinze:** Formal analysis, Investigation, Writing – review & editing. **Denny Pellowski:** Formal analysis, Investigation, Writing – review & editing. **Elisa Richter:** Formal analysis, Investigation. **Karolin Schröder:** Formal analysis, Investigation. **Lars Dahmen:** Formal analysis, Investigation, Writing – review & editing. **Christoph Schüßler:** Formal analysis, Investigation, Writing – review & editing. **Kostja Renko:** Formal analysis, Investigation, Writing – review & editing. **Tanja Schwerdtle:** Conceptualization, Writing – review & editing. **Hajo Haase:** Methodology, Writing – review & editing. **Anna P. Kipp:** Conceptualization, Funding acquisition, Supervision, Writing – original draft, Writing – review & editing.

## Declaration of competing interest

The authors declare the following financial interests/personal relationships which may be considered as potential competing interests:Anna Kipp reports financial support was provided by German Research Foundation. Anna Kipp reports financial support was provided by Carl Zeiss Foundation. Anna Kipp reports financial support was provided by Free State of Thuringia and the European Social Fund. Anna Kipp reports a relationship with Food Federation Germany that includes: speaking and lecture fees. Anna Kipp reports a relationship with BioSyn Arzneimittel GmbH that includes: speaking and lecture fees. If there are other authors, they declare that they have no known competing financial interests or personal relationships that could have appeared to influence the work reported in this paper.

## Data Availability

Data will be made available on request.
