# Supplementary material for: Distribution interactions of the trace elements zinc, copper, and selenium under conditions of their parallel deficiency
Source: Redox Biol. 2025 Dec 6;89:103963. doi: 10.1016/j.redox.2025.103963 (PMC12767715; doi:10.1016/j.redox.2025.103963)
Supplement: Multimedia component 1 [file mmc1.docx]

**Distribution interactions of the trace elements zinc, copper, and selenium under conditions of their parallel deficiency**

Kristina Lossow^1,2^, Maria Maares^2,3,4^, Tom Heinze^2,4^, Denny Pellowski^2,4^, Elisa Richter^1^, Karolin Schröder^1^, Lars Dahmen^5^, Christoph Schüßler^4^, Kostja Renko^5^, Tanja Schwerdtle^2,6^, Hajo Haase^2,3^, Anna P. Kipp^1,2^

^1^Department of Nutritional Physiology, Institute of Nutritional Sciences, Friedrich Schiller University Jena, Jena, Germany

^2^TraceAge-DFG Research Unit on Interactions of Essential Trace Elements in Healthy and Diseased Elderly, Potsdam-Berlin-Jena, Germany

^3^Department of Food Chemistry and Toxicology, Technische Universität Berlin, Berlin, Germany

^4^Department of Food Chemistry, Institute of Nutritional Science, University of Potsdam, Golm, Germany

^5^German Federal Institute for Risk Assessment (BfR), Berlin, Germany

^6^Max Rubner-Institut, Karlsruhe 76131, Germany

Correspondence to: Anna P. Kipp; postal address: Friedrich Schiller University Jena, Institute of Nutritional Sciences, Department of Nutritional Physiology, Dornburger Str. 24, 07743 Jena, mail: anna.kipp@uni-jena.de

ORCID: Anna Kipp 0000-0002-3307-1038

**Supplementary material**

**Table S1. Relative copper zinc ratio in various murine organs.** Relative ratio of copper to zinc concentrations in serum and various organs of 19-weeks-old male and female C57BL/6Jrj mice (n = 8-9) receiving a diet with varying levels of the trace elements copper, zinc and selenium. The ratio is expressed as fold change compared to controls for each sex. Blue indicates a downregulation and red an upregulation in comparison to +TE.

**Figure S1.**

**
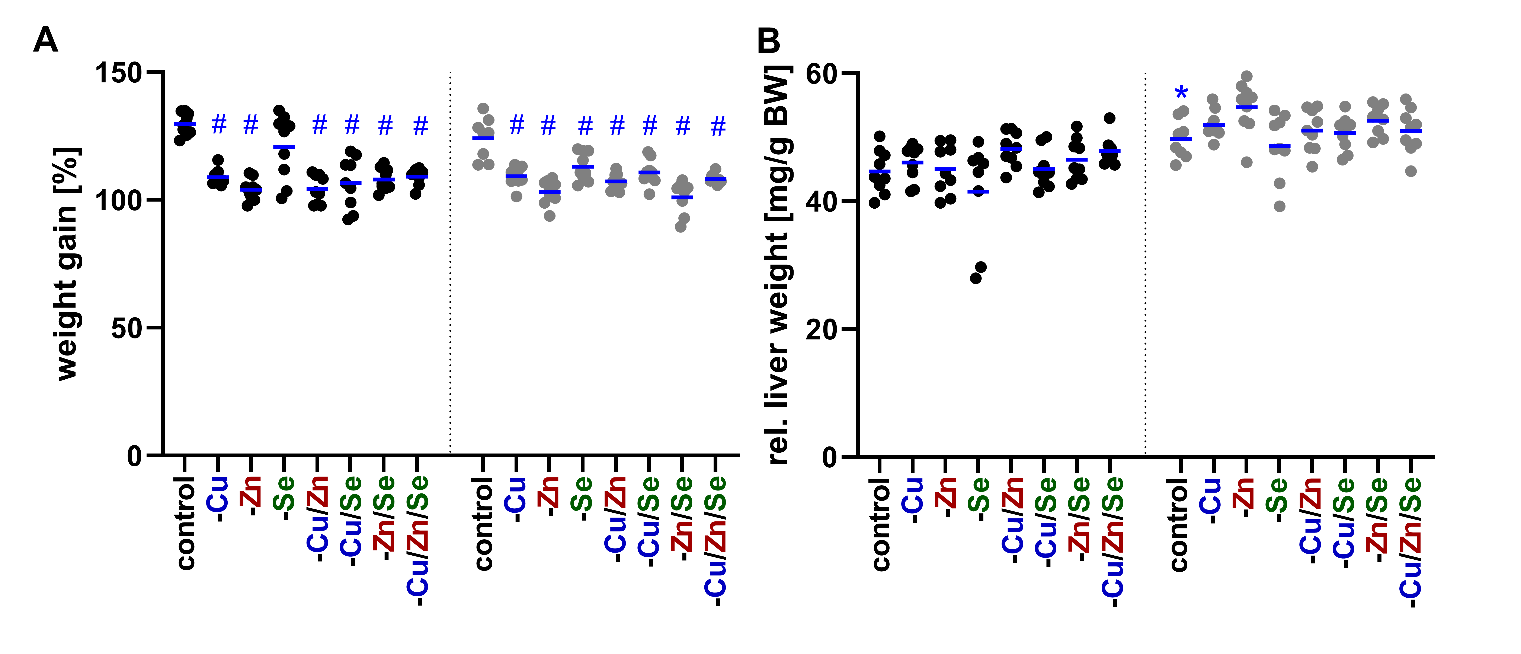
**

**Figure S1. Characteristics of the C57BL/6Jrj mice after eight weeks of dietary intervention with insufficient copper, zinc, and selenium supply, as well as combinations thereof.** Group-dependent analysis of weight gain (A) and liver weights (B). The latter was normalized to the body weight (BW) of the animals at the time of sacrifice. The individual measurements for each mouse and their average values are shown. The data for the males are displayed in black and the data for the females in grey. Statistical testing based on Two-Way ANOVA and Bonferroni's post-test with ^#^p < 0.05 compared to sex-specific control and *p < 0.05 compared to male control (only shown for adequately fed animals).

**Figure S2.**

**
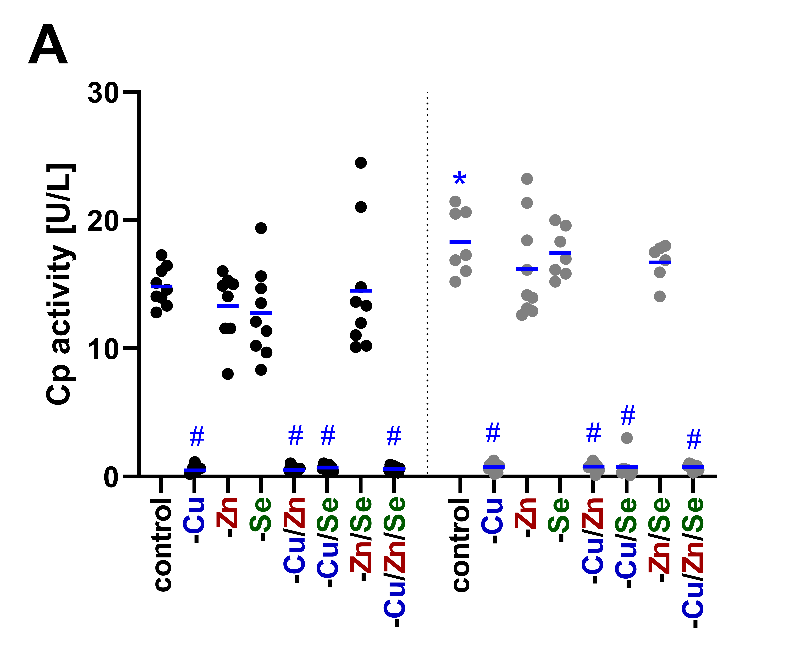
**

**Figure S2. Changes of copper-dependent enzyme activity in serum of C57BL/6Jrj mice.** The copper status was validated by serum Cp activity, based on the conversion of O-dianisidine, in the serum of 19 weeks old male and female C57BL/6Jrj mice (n = 8-9) receiving a diet with varying trace element supply. The individual measurements for each mouse and their mean values are shown. The data for the males are displayed in black and the data for the females in grey. Statistical testing based on Two-Way ANOVA and Bonferroni's post-test with ^#^p < 0.05 compared to sex-specific control and *p < 0.05 compared to male control (only shown for adequately fed animals).

**Figure S3.**


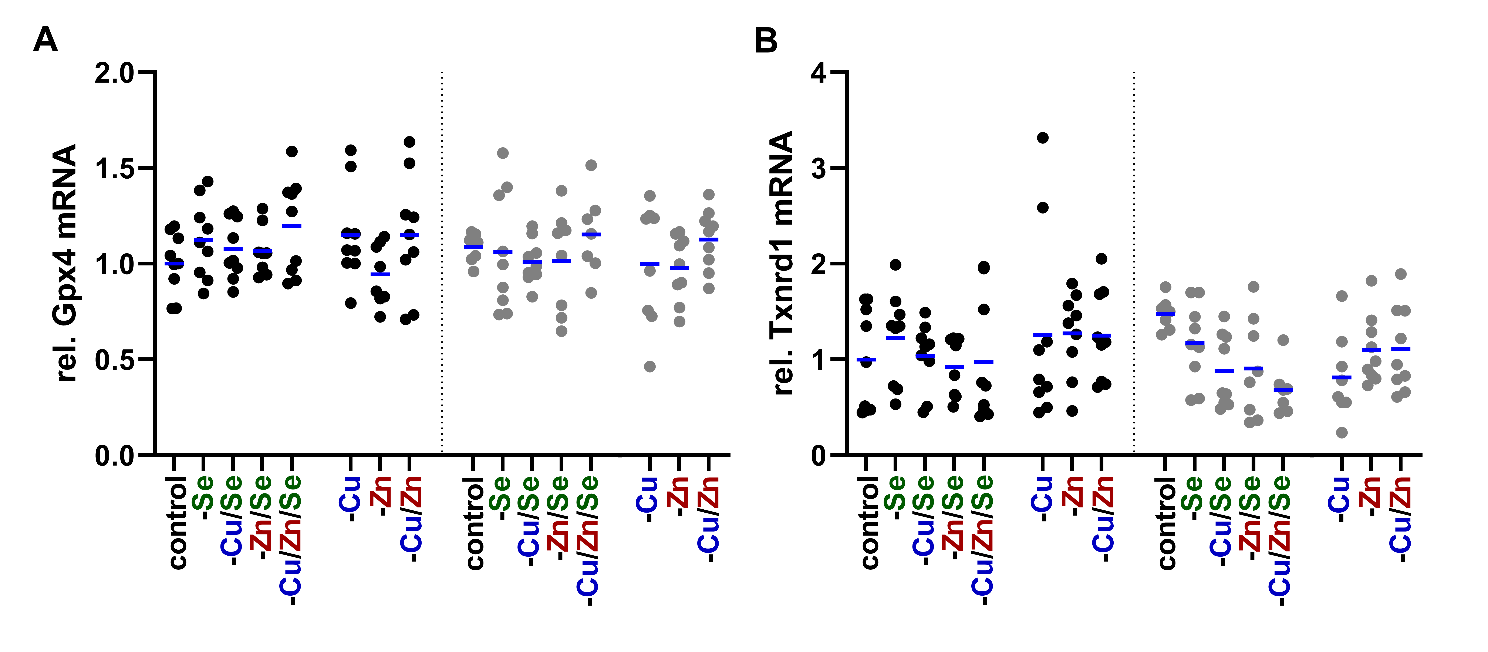


**Figure S3. Relative expression of selenoproteins in liver of C57BL/6Jrj mice.** Relative expression levels of selenoproteins like glutathione peroxidase 4 (Gpx4; A) and thioredoxin reductase 1 (Txnrd1; B) were determined by qRT-PCR in liver tissue of 19-weeks-old male and female C57BL/6Jrj mice (n = 8-9) receiving a diet with varying levels of the trace elements copper, zinc, and selenium. Expression levels were normalized to a composition factor based on Hprt, Rpl13a, and Rpl37. All results are expressed as fold change compared to male controls. Data for the males are displayed in black and for the females in grey. Statistical testing based on Two-Way ANOVA and Bonferroni's post-test with ^#^p < 0.05 compared to sex-specific control and *p < 0.05 compared to male control (only shown for adequately fed animals).

**Figure S4.**

**
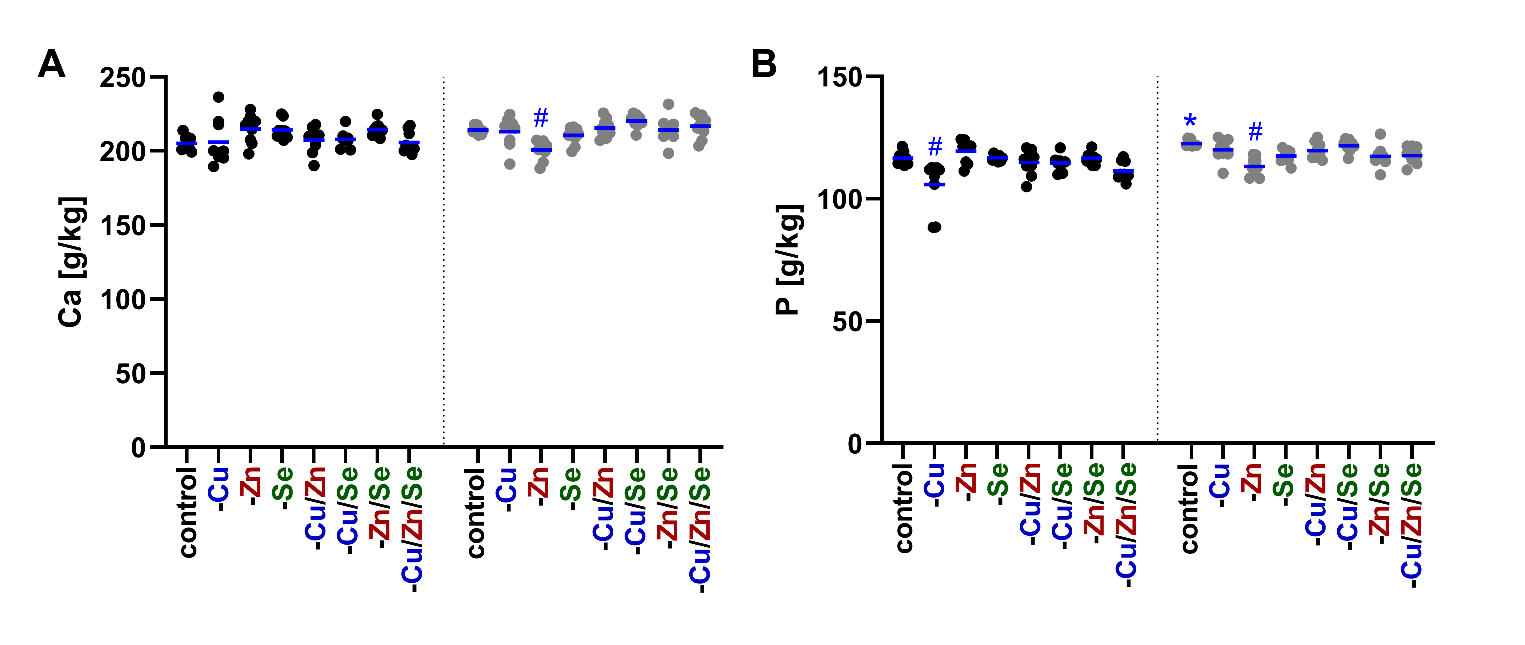
**

**Figure S4. Alterations of calcium and phosphate levels in femur of C57BL/6Jrj mice.** Concentrations of calcium (Ca; A) and phosphate (P; B) were analyzed in pulverized femur of 19-weeks-old male and female C57BL/6Jrj mice (n = 8-9) receiving a diet with varying levels of these trace elements. Trace element concentrations were determined using ICP-MS/MS after microwave-assisted digestion, normalized to whole bone dry weight. The individual measurements for each mouse and their mean values are shown. The data for the males are displayed in black and the data for the females in grey. Statistical testing based on Two-Way ANOVA and Bonferroni's post-test with ^#^p < 0.05 compared to sex-specific control and *p < 0.05 compared to male control (only shown for adequately fed animals).
